# miR-146 and miR-155: Two Key Modulators of Immune Response and Tumor Development

**DOI:** 10.3390/ncrna3030022

**Published:** 2017-06-26

**Authors:** Ugo Testa, Elvira Pelosi, Germana Castelli, Catherine Labbaye

**Affiliations:** Department of Hematology, Oncology and Molecular Medicine, Istituto Superiore di Sanità, Viale Regina Elena 299, 00161 Rome, Italy; elvira.pelosi@iss.it (E.P.); germana.castelli@iss.it (G.C.); catherine.labbaye@iss.it (C.L.)

**Keywords:** miR, immunity, cancer

## Abstract

MicroRNAs (miRNAs or miRs) are a class of evolutionarily-conserved small, regulatory non-coding RNAs, 19–3 nucleotides in length, that negatively regulate protein coding gene transcripts’ expression. miR-146 (146a and 146b) and miR-155 are among the first and most studied miRs for their multiple roles in the control of the innate and adaptive immune processes and for their deregulation and oncogenic role in some tumors. In the present review, we have focused on the recent acquisitions about the key role played by miR-146a, miR-146b and miR-155 in the control of the immune system and in myeloid tumorigenesis. Growing experimental evidence indicates an opposite role of miR-146a with respect to miR-155 in the fine regulation of many steps of the immune response, acting at the level of the various cell types involved in innate and adaptive immune mechanisms. The demonstration that miR-155 overexpression plays a key pathogenic role in some lymphomas and acute myeloid leukemias has led to the development of an antagomir-based approach as a new promising therapeutic strategy.

## 1. Introduction

MicroRNAs (miRNAs or miRs) are a family of 19–23-nucleotide non-coding RNAs that play a key role in multiple biological processes through the regulation of the levels of many proteins [1]. This function is exerted at the level of the mRNA 3′ untranslated regions (3′-UTR) to suppress their translation. In addition to the suppression of mRNA translation, miRs may control mRNA degradation and mRNA decay. miRs bind to the 3′-UTR of target mRNAs by direct base-pairing to the 5′-miRNA seed region, resulting either in repression of mRNA translation or induction of mRNA degradation. These RNAs are transcribed as primary large transcripts miRNAs (pri-miRNAs) and are processed in the nucleus by the complex formed by RNAse III endonuclease enzyme DROSHA and the RNA-binding protein DGCR8, to generate a ≈85-nucleotide stem loop, called the precursor miRNAs (pre-miRNAs); these precursors are then exported from the nucleus to the cytoplasm by the exportin 5/RanGTP complex (XPO5); in the cytoplasm, another endonuclease enzyme, DICER, cleaves the precursor miRNAs, generating a 20–22-nucleotide mi-R/miR* duplex (formed by the mature strand and the “passenger” strand) and then the mature miRNAs. A recent study re-evaluated the role of DROSHA, DICER and XPO5 in miRs biogenesis, providing evidence that DROSHA is essential, DICER is important, while the function of XPO5 is not strictly necessary, since it can be complemented by alternative mechanisms [2].

More than 1000 conserved miR are encoded by the mammalian genome. Currently, there is a total of 1881 annotated human miR precursor genes [3], but the function of many of these miRs is largely unknown. They regulate many biological processes, including development, differentiation, metabolism and cellular physiology.

Importantly, miRs have been shown to function as key regulators of immune responses under both normal and pathological conditions [4,5]. Some miRs play a crucial role as fine regulators of the innate and adaptive immune system, and some of them act as critical modulators of the immune response against tumors [4,5]. 

**Figure 1:**
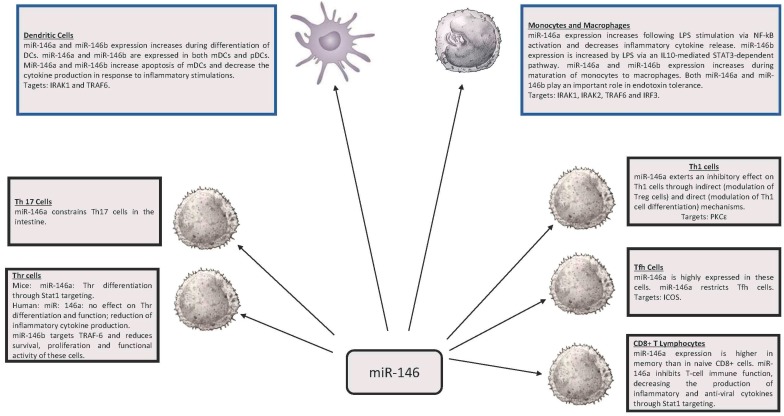
Expression and function of miR-146 in various cell types of the immune system. mDC: myeloid Dendritic Cell. pDC: plasmocytoid Dendritic Cell. PLPS: lipopolysaccharide.

**Figure 2:**
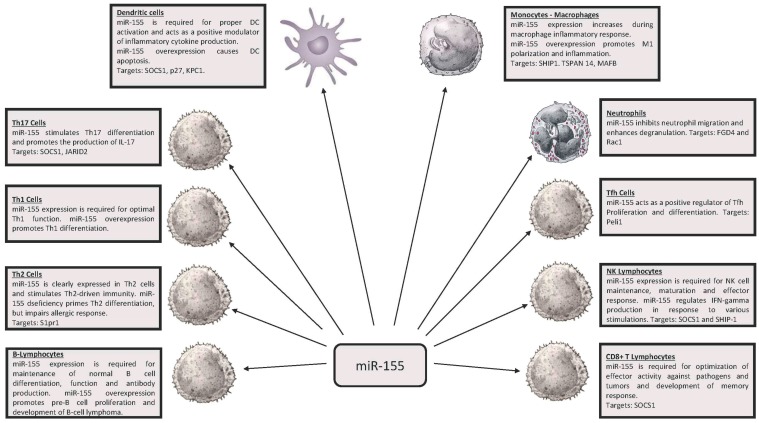
Expression and function of miR-155 in various cell types of the immune system. DC: Dendritic Cell. NK: Natural Killer.

Among the various miRs playing a possible role in the control of the immune response, miR-146 (miR-146a and miR-146b) and miR-155 emerged as key regulators of the immune system (Figure 1 and Figure 2); a deregulated expression of these miRNAs is involved also in development of some cancers. miR-146 and miR-155 are conserved in their sequence in the evolution of vertebrate species, but do not show sequence similarity. These miRs arise from different genetic loci and are generated via different routes of RNA processing: miR-146a from a pre-mRNA; miR-146b from an mRNA intron; miR-155 from an exon of a long non-coding RNA. 

Both miR-146a and miR-155 are particularly responsive to many inflammatory stimuli, as those induced by some cytokines (TNFα, IL-1β, type I and type II interferons (IFNs) or RANKL) or to Toll-like receptor (TLR) ligand in various cell types, and particularly in monocytes/macrophages (reviewed in [6] and [7]). Both of these miRs target and repress several TLR4 effectors, such as TNFα, PU.1, SHIP1, SOCS1 (miR-155), TNFR-associated factor 6 (TRAF6), IRAK1, IRAK2, IRF3 and IRF5 (miR-146a). Finally, the expression of both of these miRNAs has been associated with various pathologic conditions, characterized by chronic inflammation, such as rheumatoid arthritis, systemic lupus erythematosus, periodontitis, nephropathy and atherosclerosis.

**Figure 3:**
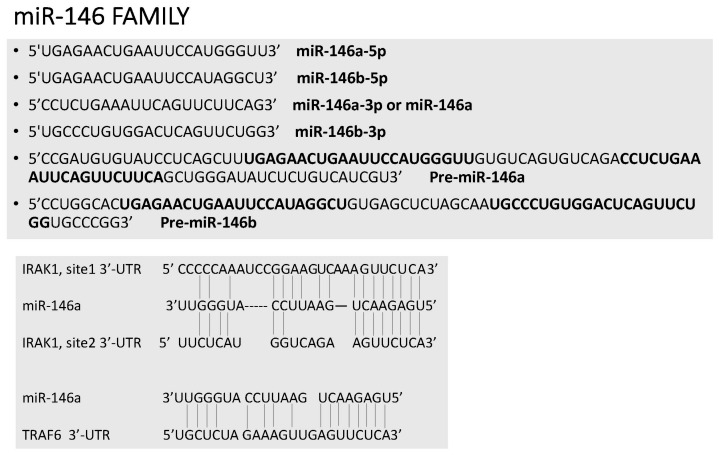
miR-146 family. Top panel: Sequence of pre-miR-146a and pre-miR-146b, mature miR-146a-5p, miR-146b-5p, miR-146a-3p and miR-146b-3p. Bold letters correspond to the sequence of mature miRs. Bottom panel: Molecular targets of 146a: IRAK1 and TRAF6.

The human genome contains two miR-146 genes (miR-146a and miR-146b) on chromosomes 5 and 10, respectively. Each pre-miR generates two mature miRs: miR-146a-5p and miR-146a-3p; miR-146b-5p and miR-146b-3p (Figure 3). miR-146a-5p and miR-146b-5p are the most characterized and differ only by 2 nt in the 3′ region. Importantly, miR-146a-5p and miR-146b-5p, here called miR-146a and miR-146b, share a seed sequence and are, therefore, predicted to target the same set of genes. Gene knockout studies have shown that miR-146a deficiency leads to an excessive IL-6 and TNFα production, myeloproliferative syndrome, chronic inflammation and a decrease in the number and quality of hematopoietic stem cells [8,9]. In the absence of miR-146a expression, regulatory T lymphocytes (Treg) lose their suppressive effects, as a consequence of Stat1 overexpression, inducing decreased IFN-γ secretion [10]. According to these findings, dysregulated miR-146a expression is observed in autoimmune disease.

**Figure 4:**
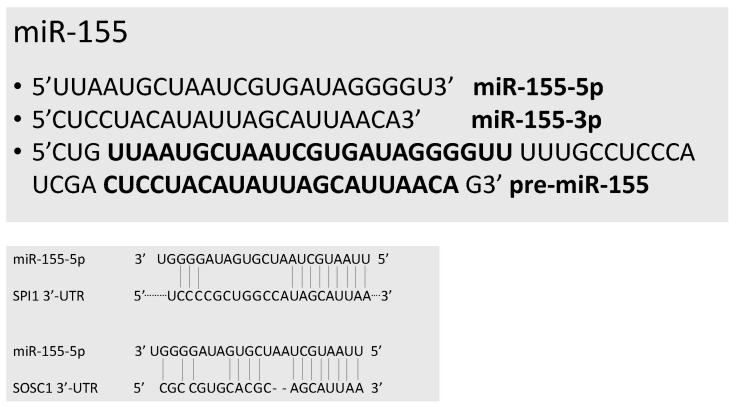
miR-155. Top panel: Sequence of pre-miR-155, mature miR-155-5p and miR-155-3p. Bold letters correspond to the sequence of mature miRs. Bottom panel: Molecular targets of miR-155: SPI1 and SOCS1.

miR-155 was originally identified as a B cell integration cluster (Bic), responsible for leucosis due to its activation in the chicken genome by viral promoter insertion [11]. A homologous gene to bic in humans was subsequently identified [12]. Pre-miR-155 originates two mature miRs: miR-155-5p (here, commonly referred to as miR-155) and miR-155-3p (Figure 4). miR-155 was found to be mainly expressed in the thymus and in the spleen. miR-155 was one of the miRs specific for the hematopoietic system (miR-142, miR-144, miR-150, miR-155 and miR-223). Subsequent studies have supported that miR-155 could represent an oncomiR, whose expression is activated in many tumors, particularly those of the lymphoid tissue [13,14]. Mice deficient for miR-155 are viable and do not show any macroscopic developmental defect. No gross defects in myeloid or lymphoid development in miR-155-deficient mice were observed; however, miR-155-deficient mice displayed a defect in protective immunity when exposed to a challenge with infective agents, due to a defective function of B, T and dendritic cells [15]. This fundamental study thus indicated a key role of miR-155 as a modulator of immune function.

## 2. miR-146, miR-155 and Innate Immunity

A set of studies has supported a role for miR-146a in the control of the biological functions of antigen-presenting cells. Two types of cells act as antigen-presenting cells: dendritic cells (DCs) and monocytes (Mos). Both DCs and monocytes are heterogeneous, with different populations displaying different effector and immunomodulatory functions. Particularly, two different sets of human circulating DCs have been identified: myeloid (mDCs or DC1) and plasmacytoid (pDCs or DC2) dendritic cells. mDCs can be subdivided into two different subsets distinguished according to membrane markers and functional properties: a major CD1c^+^ subset, highly effective in antigen uptake, migration and antigen presentation to CD4^+^ T lymphocytes (these DCs promote Th17- and Th2-mediated immune response to extracellular pathogens) and a minor CD141^+^ subset, specialized in antigen cross-presentation and in CD8^+^ T cell priming for tumors and pathogens. pDCs are a subset of DCs that secrete type I IFN in response to viruses. pDCs express CD123 and TLR7 and TLR9, represent the first line of host defense against viruses and bacteria and link innate to adaptive immunity. Langerhans cells (LCs) are mDCs localized within epidermis, mucosal or bronchial epithelium, express CD1a and can be activated by local inflammatory stimulation, migrating to driving lymph nodes and differentiating into interdigitating cells (IntDCs). Similarly, human peripheral blood monocytes are heterogeneous and consist of three different subpopulations distinguished according to CD14 and CD16 Fcγ receptor expression: (a) a CD14^++^/CD16^−^ population, defined also as classical monocytes (cMo), representing the large majority of peripheral blood (PB) monocytes (90–95%); (b) CD14^low/−^/CD16^+^ non-classical monocytes; (c) CD14^+/low^/CD16^low^ or intermediate monocytes (iMo).

DC subsets are originated through the differentiation of a common progenitor, the monocyte and DC progenitor: this progenitor generates a common DC progenitor and monocytes [16]. Common DC progenitors are present in bone marrow, where they generate pDCs and distinct circulating progenitors pre-committed to become CD1c^+^ or CD141^+^ DCs [17,18].

Few studies have explored miR-146 expression in DCs. An initial study showed that miR-146 expression was lower in dendritic cells originated from monocytes grown in the presence of IL-4 and GM-CSF, compared to dendritic cells originated by cultivation of monocytes in the presence of IFN-α [18]. The lower miR-146 expression in these cells is accompanied by an increased migratory capacity and NF-ĸB expression [19]. miR-146a and particularly miR-146b expression were markedly up-modulated during maturation of monocytes to dendritic cells using GM-CSF and IL-4: particularly, miR-146a and miR-146b expression was about 10–40-fold, respectively, higher than in monocytes [20]. In a more recent study, the same authors explored the functional role of miR-146a and miR-146b in human dendritic cells. First, it was confirmed that both miR-146a and miR-146b expression markedly increased during dendritic cell differentiation from monocytes; both the miRs efficiently target in these cells TRAF-6 and IRAK-1 and, through this mechanism, increase the spontaneous apoptosis of dendritic cells; miR-146a and miR-146b do not affect dendritic cell maturation, but modulate the production of pro-inflammatory cytokines in mature dendritic cells [21]. These observations suggest a role for miR-146a and miR-146b in the regulation of dendritic cell survival and inflammatory activation [21]. 

Other studies were focused on analyzing miR-146 levels in dendritic subsets. Thus, Jurkin and coworkers have shown that plasmacytoid DCs express miR-146a at levels four-times higher than myeloid int-DCs [22]. pDCs constitutively express miR-146a levels much higher than mature monocytes: however, miR-146a expression is strongly activated in monocytic cells, but not in pDCs by IFN-γ, IL-1β and lipopolysaccharide (LPS) [22]. miR-146a expression is up-modulated during differentiation of pDCs by the transcription factor PU.1, and by itself, miR-146a was unable to affect DC differentiation [19]. miR-146a expression in pDCs is markedly up-modulated upon activation of primary pDCs using TLR7 or TLR9 agonists [23]. miR-146a is functionally involved in the regulation of the survival and TLR-induced maturation of pDCs [23]. 

miR-155 expression is required for proper DC activation, probably through the regulation of the suppressor of cytokine signaling 1 (SOCS1) protein levels [23,24]. In fact, DCs lacking miR-155 are defective at inducing T cell activation [24]. In DCs, miR-155 seems to act as a positive modulator of inflammatory cytokine production [25]. Particularly, miR-155 up-modulates the IL-1 signaling pathway in monocyte-derived DCs [26]. miR-155 targets the DC-specific intracellular adhesion molecule-3 grabbing non-integrin (DC-SIGN) and reduces its pathogen binding capacity during maturation [26]. Experiments of neutralization of miR-155 expression in human monocytes maturing to dendritic cells have shown that miR-155 expression is not required for dendritic cell differentiation, but is required for IL-12 expression; SOSC1 and M-CSFR were shown as functional targets of miR-155 in these cells [27]. Few studies have explored the functional role of miR-155 in vivo. miR-155 upregulation in DCs targets SHIP1, and the SHIP1 down-modulation in DCs is sufficient to break T cell tolerance in vivo and to promote a CD8-mediated autoimmune response in vivo [28]; conversely, DCs lacking miR-155 have an impaired ability to break immune tolerance [28].

Several recent studies have shown a relevant role of miR-146 in the control of the natural immune activity of monocytes/macrophages. In this context, a key observation was made by Taganov and coworkers in 2006, reporting the expression profiling of 200 miRNAs in human monocytes stimulated with bacterial LPS; among them, miR-146a, and to a lesser extent, also miR-146b are rapidly and markedly induced by bacterial LPS stimulation, and this induction is dependent on the transcription factor NF-ĸB [29]. Paradoxically, miR-146a acts as a negative regulator of NF-ĸB: in fact, miR-146a induces a reduced phosphorylation of IĸBα at the level of serine 32, a factor essential for its degradation and release from the IĸBα/NF-ĸB complex. More specifically, the experiments carried out in LPS-stimulated monocytic cells have shown that miR-146a targets IRAK1, IRAK2 and TRAF6, reducing their expression, all molecules playing a key role as adaptor kinases of the MyD88-dependent signature pathway, induced by the activation of the Toll like receptor 4 (TLR4); in addition, miR-146a targets also IRF3, another component of the MyD88-dependent signaling pathway [20,29]. According to these findings, it was proposed that miR-146a controls TLR4 signaling through a regulatory loop, involving the upregulation of miR-146a expression by activated NF-ĸB; in turn, miR-146a targets and reduces the expression of TRAF6, IRAK1, IRAK2 and IRF3, thus resulting in reduced activity of both NF-ĸB and IRF3.

miR-146a expression increases during the differentiation of monocytes to macrophages: LPS increases miR-146a, while IL-4 reduces miR-146a expression during macrophagic maturation [30].

LPS stimulation induces an increase not only of miR-146a, but also of miR-146b. However, the miR-146b increase is mediated by an IL-10-dependent STAT3-regulated loop [31] and induces an anti-inflammatory effect related to the targeting of TLR4, IRAK1, TRAF6 and MyD88; miR-146b overexpression in macrophagic cells determines a marked reduction of LPS-dependent production of inflammatory cytokines and chemokines [31]. Subsequent studies have explored the possible physiological role of miR-146b induction elicited by IL-10. IL-10 is a strong anti-inflammatory cytokine playing an important role as an IFN-γ suppressing factor acting on Th1 cells. Interestingly, in IL-10-deficient mice, miR-146b expression is deficient in macrophages [32]. miR-146b targets IRF5 and, through this mechanism, exerts an inhibitory effect on macrophage activation; in line with this finding, miR-146b-deficient mice develop intestinal inflammation with enhanced M1 macrophage activation [32]. The ensemble of these studies supports a physiological role for miR-146b in the control of macrophage activation [32]. Interestingly, miR-146b^-/-^ mice displayed enlarged spleens, increased myeloid cell populations both in spleen and bone marrow and spontaneously develop intestinal inflammation [32].

In parallel, other studies have reported the induction of miR-155 expression during the macrophage inflammatory response induced by various stimuli, including TLR ligands, IFN-beta and IFN-gamma [33]. miR-155 activation involves the c-Jun N-terminal Kinase (JNK) pathway [34]. An important target of miR-155 in activated macrophages is represented by inositol phosphatase SHIP1, resulting in the consequent increase of AKT kinase activity [35].

Following different types of environmental stimulations (cytokines, pathogens), macrophages develop different phenotypes and functions. These various macrophagic phenotypes can be mimicked in vitro through the stimulation of monocytes with LPS and IFN-γ inducing classical inflammatory M1 macrophages or IL-4 inducing the generation of M2 macrophages. The generation of M1 macrophages is associated with a high induction of miR-155 expression, while the generation of M2 macrophages is associated with a markedly lower miR-155 induction [36]. Experiments of miR-155 overexpression or depletion supported a functional role for miR-155 in the programming of macrophages to pro-inflammatory M1 macrophages [37]. Studies carried out in miR-155 knockout mice support a key role of miR-155 in the control of the inflammatory signature observed in M1 macrophages [14]. In addition to SHIP1, other relevant targets of miR-155 in these cells are represented by TSPAN14, INPP5D and MAFB [14].

miR-155 and miR-146a play a key role in endotoxin tolerance. Endotoxin tolerance is a state in which macrophages that have been previously exposed to bacterial LPS, develop a refractivity to an additional stimulation. Endotoxin tolerance plays a key physiopathological role in the context of diseases, such as sepsis and trauma, and exerts a protective effect, limiting the negative consequences of an excessive inflammation and of endotoxin shock. AKT signaling plays a key role in the development of endotoxin tolerance. AKT1 is indispensable for endotoxin tolerance generation and acts through a mechanism involving the suppression of miR-155 and consequent up-modulation of its target SOCS1, induction of let-7e and reduced TLR4 expression [38]. Furthermore, AKT1 regulates the expression of both miR-146a and miR-155 in a coordinated way, mediating molecular signals that determine co-localization and silencing of one of their genomic loci, through this mechanism maintaining low levels of expression and inhibiting upon a subsequent stimulus [39]. Other studies support a role for miR-146a in endotoxin tolerance: transfection of a miR-146a inhibitor in monocytic cells abolished LPS tolerance [40]. In vitro studies using monocytic cells have shown the key role of miR-146a in mediating endotoxin tolerance to lipo-oligosaccharides of pathogenic *Neisseria* bacteria [41]. The important role of miR-146a in endotoxin tolerance is also supported by the analysis of the effects of morphine on endotoxin tolerance: chronic morphine treatment tempers endotoxin tolerance, resulting in persistent inflammation, septicemia and septic shock; this drug consistently downregulates LPS-induced miR-146a and miR-155 in macrophages [42]. miR-146a overexpression abrogates morphine-mediated hyper-inflammation [42]. Finally, miR-146a was found to be indispensable for the development of endotoxin tolerance to intestinal bacteria in neonates [43].

It is important to point out that increased miR-146a levels observed in sepsis may contribute to the attenuation of cardiac dysfunction observed in sepsis, acting at the level of cardiomyocytes and of macrophages present in cardiac tissue [44]. 

Several studies have defined a possible physiopathological role of miR-146a and miR-155 in bacteria-related or inflammatory processes involving macrophages as a main mechanism of innate immunity defense.

Particularly interesting are the observations reported in *Mycobacterium tuberculosis* (MTB) infection. Monocytes/macrophages represent the first line of defense against MTB. During MTB infection, invading bacteria are recognized by macrophages and induce in these cells adaptive immune responses; TLRs are involved in the recognition of MTB and are consequently activated and, via MyD88 signaling, activate NF-ĸB and MAPK pathways, resulting in the production of inflammatory cytokines and anti-microbial mediators, such as TNF-α and nitric oxide (NO) [45]. 

Initial studies have shown that several miRNAs, including miR-155, miR-146a and miR-132, are induced in macrophages during MTBinfection [46]. In a subsequent study, the same investigators have analyzed the consequences of miR-146a induction by MBT: miR-146a induced by MBT in macrophages attenuates iNOS and NO production in infected macrophages by targeting TRAF6 and, through this mechanism, dampens host defense against intracellular bacteria, such as MBT [47]. Other studies have confirmed these findings showing that miR-146a expression was increased in a dose-dependent manner in MBT-infected macrophages: the increased miR-146a expression reduces the induction of pro-inflammatory cytokines, such as TNF-α, IL-1β, IL-6 and MCP-1, via the IRAK-1 and TRAF-6 targeting; these findings indicate that miR-146a up-modulation exerts an anti-inflammatory effect and, in line with this interpretation, a higher bacterial burden was observed in miR-146a mimics-treated macrophages [48]. Other studies have shown that the capacity to induce miR-146a expression is displayed by both pathogenic and non-pathogenic strains of MBT [49].

Few studies have explored miR-146a expression in monocytes/macrophages directly derived from MBT patients and not challenged in vitro with living bacteria. Two studies showed a decreased expression of miR-146a at the level of either peripheral blood monocytes or macrophages present in bacterial lavage fluids [50,51]. Interestingly, miR-146a levels observed in Peripheral Blood Mononuclear Cells (PBMCs) increased following anti-mycobacterial therapy [50]. 

It is of interest to compare the results observed for miR-146a with those found for miR-155, another miR involved in the control of immune-related genes. miR-155 expression is transiently induced in human monocytes/macrophages following infection with mycobacterium tuberculosis. Initial studies examining the role of miR-55 in regulating macrophage responses to mycobacteria in vitro have generated highly variable and discordant results, probably related to the use of virulent and avirulent mycobacteria strains and to monocytic cell lines or primary monocytic/macrophagic cells [46,52,53,54,55]. Experiments carried out using the virulent MBT strain support a permissive role for miR-155 in the infection of macrophages by mycobacteria: in fact, miR-155 maintains the survival of MBT-infected macrophages (through an inhibitory effect on SH2 domain-containing inositol 5-phosphatase 1 (SHIP1) and a consequent increase of phospho-AKT), providing a cellular niche favoring bacterial replication [55]. However, the impact of miR-155 on mycobacterium infection is more complex in that this miR exerts also important effects on the adaptive response of T cells to mycobacteria. Thus, while miR-155 expression at the level of macrophages creates conditions favorable for mycobacterium survival, at the level of T cells, this miR favors the development of adoptive T cell responses to mycobacteria [55]. In line with these conclusions, miR-155^−/−^ mice exhibit early resistance to mycobacteria (due to a reduced growth of bacteria in infected monocytes/macrophages), but an increased susceptibility to mycobacterium during the chronic stage of infection (due to reduced T cell immunity). 

## 3. miR-146 and miR-155 in Adaptive Immunity

miR-146 and miR-155 play an important role also in some processes related to adaptive immunity.

### 3.1. Regulatory T Lymphocytes (Treg) 

Several studies have supported an important role of miR-146 and miR-155 in the control of Treg cells. These CD4^+^ lymphocytes, characterized by the expression of the transcription factor Foxcp3, exhibit a repressive function on the activity of effector T lymphocytes. Their biologic activity is essential for a fine modulation and tuning of the immune response. miR-146a is highly expressed in Treg cells, and its expression in these cells is essential for the capacity of Treg cells to restrain IFN-γ-mediated pathogenic Th1 and associated inflammatory response. Particularly, in Treg cells, the suppressive effect of miR-146a on Stat1, a transcription factor required for Th1 differentiation, is strictly necessary for the repressive functional activity of Treg on Th1 responses [10]. miR-146a and miR-146b, together with miR-27, miR-660, let-7g and miR-598, are the only miRs clearly more expressed in Treg than in Tconvs [56]. Since miR-146a expression is particularly pronounced in Treg cells, the detection of miR-146a and miR-142-3p was proposed as a potential biomarker of human Treg cells [57]. It is important to note that some studies carried out in human Treg cells have failed to show an effect of miR-146a favoring Treg cell differentiation and function. Thus, Bhairababholta and coworkers failed to show that miR-146a silencing affects the biologic activity of human Treg, as assayed by conventional in vitro assays [56]. Zhou et al., through the study of Treg in arthritis rheumatoid patients, reached the conclusion that miR-146a controls the inflammatory phenotype of these cells, but is unable to modify their Treg function [58]. They observed a diminished miR-146a expression in Treg cells of these patients, particularly pronounced in patients with active disease and marked joint inflammation [58]. Up-modulation or silencing of miR-146a expression, with mimics or antagomirs, in purified Treg, modified cytokine secretion, but failed to change the Treg function [57]. A recent study explored the expression and function of miR-146b in human Treg and obtained evidence suggesting a functional role different from that described through the study of miR-146 knockout mice. This study showed that miR-146b is one of the miRNAs more expressed in Treg cells; the abrogation of its expression with a specific antagomir enhanced TRAF6 expression and NF-ĸB activity and resulted in increased survival, proliferation and functional activity of Treg cells; surprisingly, blocking of miR-146a expression failed to induce these effects [59]. According to these findings, it was concluded that an miR-146b/TRAF6/NF-ĸB/FoxP3 pathway restrains Treg cell survival and activity [59]. 

The study of the effect of miR-155 on Treg cells generated controversial results. The immunological suppressive activity of Treg cells remained unchanged in miR-155-deficient mice [60]; however, the number of Treg cells in the spleen and in the thymus of miR-155^−/−^ mice is reduced [24,60]. FoxP3 (a transcription factor essential for Treg differentiation) is stimulated by miR-155, through the activation of SOCS1, increasing the activation of STAT5 and its binding to the FoxP3 promoter; in turn, Foxp3 increases the expression of miR-155 [24,59]. Recently, it was shown that miR-155^−/−^ mice have a reduced number of CD69^+^ Treg cells; CD69 was shown to be required for the development of Treg cells in the thymus through the promotion of STAT5 and the transcription of miR-155 [61].

While the role of miR-155 in the regulation of Treg cell differentiation seems to be more defined, its role in regulating Treg suppressive function is less clear. In this context, Zhou et al., investigating Treg cells of rheumatoid arthritis patients, reached the same conclusion as for miR-146a: the level of miR-155 expression regulates the cytokine secretion pattern, but not the immunosuppressive capacity of Treg cells [58].

### 3.2. Th1 and Th2

As mentioned above, the study of miR-146a^-/-^ mice has shown that miR-146a exerts a negative control on Th1 function mainly through an indirect mechanism related to a stimulatory effect on Treg immunosuppressive function [9]. Furthermore, miR-146a was identified as a regulator of the resolution of T cell responses, exerting a negative control on T cell receptor-mediated NF-ĸB activation [62]. The study of human Th1 cells showed the existence of a new direct molecular pathway through which miR-146a exerts an inhibitory effect on the differentiation of these cells. In fact, it was shown that miR-146a targets protein kinase Cε (PKCε), forming a complex with Stat4, which acts as a driver of Th1 cell differentiation [63]. Sepsis patients display reduced levels of miR-146a, associated with increased PKCε levels, a condition that exacerbates the initial hyperinflammatory phase of the disease and promotes an excessive Th1 response [63]. 

miR-155^−/−^ mice displayed normal Th1 and Th2 cell levels. However, various defects in CD4^+^ T cells present in miR-155^−/−^ animals were observed: reduced IFN-γ production following activation, increased CCL-5 production by Th1 cells, increased tendency to generate Th2 cells (with increased production of the Th2 cytokines IL-4, IL-5 and IL-10) after culture in polarizing conditions [15]. These observations were interpreted as evidence supporting that miR-155-deficient CD4^+^ T cells are intrinsically biased toward Th2 differentiation [14]. Through the study of in vitro cultures of CD4^+^ T lymphocytes, Banerjee and coworkers reached the conclusion that miR-155 expression, induced in activated T cells, targets IFN-γRα and, through this mechanism, triggers Th1 differentiation [64]. Although no major phenotypic defects in Th1 cells were observed at the level of miR-155-deficient Th1 cells, however, several models of infectious diseases depending on Th1 responses have shown a role of miR-155 in Th1 cell function. Particularly, the salmonella and *Helicobacter pylori* infection models showed that bacterial eradication was impaired in miR-155-deficient mice, and this phenomenon was related to a defective Th1 response to pathogens [15,65]. Okoye and coworkers have performed a detailed analysis of miRNAs expressed in mouse Th2 T cells and provided evidence that five miRNAs (miR-15a, miR-20b, miR-146a, miR-155 and miR-200c) were preferentially expressed in these cells. Through the study of miR-155 and miR-146a knockout mice, these authors reached the conclusion that miR-155 and miR-146a exert opposing roles, promoting and regulating Th2-driven immunity (allergic and helminth-induced immunity), respectively [66]. The functional experiments provided evidence that miR-155 is required for Th2-mediated immunity. miR-155 targets several genes in Th2 cells, including S1pr1 (S1pr1 controls Th2 cell migration to the lung), preventing through this mechanism airway allergy [66].

However, more recent studies based on the analysis of allergen-challenged miR-155-deficient mice and of some allergic conditions have shown that miR-155 plays a stimulatory and not a suppressive role in the promotion of Th2 responses. In fact, Malmhall and coworkers observed a reduced allergic reaction at the level of lungs in allergen-challenged miR-155-deficient mice [67]. Thus, the ensemble of the studies performed in miR-155-deficient mice suggest that a lack of miR-155 stimulates IL-4-driven Th2 cells’ differentiation, but inhibits DC-triggered Th2 differentiation under an allergic stimulation. In line with this interpretation, miR-155 levels are markedly enhanced in the lungs of miR-155-WT allergen-challenged animals [67]. In miR-155^−/−^ animals, the lung allergic response to airway inflammation was markedly reduced, due to a reduced Th2 priming of dendritic cells [68]. Furthermore, IgE-mediated mast cell function is impaired in miR-155-deficient mice [69].

### 3.3. T Follicular Helpers (Tfh) Cells

T follicular helpers (Tfh), a subset of helper CD4^+^ T cells, are essential for the survival and selection signals to germinal center B lymphocytes, essential for the long-lived antibody production. A strict regulation of Tfh cell number and function and subsequent antibody production is a fundamental process for infection clearance, and deregulation of the mechanisms controlling Tfh responses is involved in the progression of various autoimmune diseases. miR-146a is highly expressed in Tfh cells; after an immunization, miR-146a expression reaches a peak and marks the decline of the Tfh response [70]. On the other hand, loss of miR-146a causes accumulation of Tfh and GC B-ells [70]; in Tfh miR-146a^−/−^ cells, the most upregulated gene is *ICOS* [66]. In line with this last observation, a blocking ICOS antibody prevents Tfh and Germinal Center (GC) B cell accumulation observed in miR-146a-deficient animals [70]. The study of miR-146a^−/−^ mice allowed defining a role for miR-155 in the control of Tfh cells. In fact, it was shown that miR-155 promoted Tfh cell accumulation and the increase in the number of germinal B cells and autoantibody production occurring in miR-146a^−/−^ mice displaying a chronic, low-grade inflammation [71]. According to these findings, it was proposed that miR-146a and miR-155 counter-regulate Tfh development [71]. The positive role of miR-155 in the control of the generation and function of Tfh cells was confirmed through the study of miR-155^−/−^ mice. In fact, miR-155-deficient Tfh cells display reduced cell proliferation and differentiation and decreased CD40L expression [72]. At the molecular level, miR-155 targets and reduces the expression of Peli1, a ubiquitin ligase that induces the degradation of the NF-ĸB transcription factor c-Rel, which controls CD40L expression and cellular proliferation [72].

### 3.4. CD8^+^ Lymphocytes

miR-146a expression was clearly higher in effector memory and central memory than in naive human CD8^+^ T lymphocytes [73]. miR-146a expression is greatly induced upon T cell receptor stimulation of CD8^+^ lymphocytes and reduces the spontaneous apoptosis of these cells through the targeting of the FADD apoptotic mediator [73]. According to these findings, it was suggested that miR-146a could play a role in the accumulation and/or maintenance of memory T lymphocytes [74]. miR-146a expression markedly increases in CD8^+^ lymphocytes following viral infections, as observed in patients with chronic hepatitis B: in these patients, miR-146a levels in CD8^+^ T cells correlate with necroinflammation parameters [74]. Stat1 is an important target of miR-146a in these cells, and its targeting decreases the production of anti-viral cytokines and favors viral infection [74]. In vitro blockage of miR-146a in CD8^+^ T lymphocytes significantly enhances virus-specific T cell activity. The role of miR-146a in targeting Stat1 in human T cells is also supported by the study of systemic lupus erythematosus (SLE) patients: in the PBMCs of these patients, a decreased miR-146a expression was observed, responsible for the induction of type I IFN through Stat1 and IRF5 targeting [75]. These observations support a role for miR-146a as a negative regulator of the IFN pathway in T cells.

Studies carried out in miR-155^−/−^ mice have shown that optimal miR-155 expression is required for effector CD8^+^ T cell responses to virus infection and cancer [76,77]. On the other hand, miR-155 overexpression enhanced the anti-viral, as well as anti-tumor CD8^+^ T cell responses in vivo [76,77]. SOCS1 is a key target of miR-155 in CD8^+^ T lymphocytes: in line with this finding, SOCS1 overexpression in CD8^+^ T cells phenocopied miR-155 deficiency [77]. Gene expression profiling of miR-155^−/−^ CD8^+^ T cells showed increased I IFN signaling [76]. Given the opposite effects of miR-155 and miR-146a on T cell-mediated tumor immunity, with miR-155 inhibiting tumor growth and miR-146a favoring tumor development, it seemed of interest to evaluate the effect of a double miR-155 and miR-146a knockout on T cell-mediated anti-tumor response: in these mice, the anti-tumor response, as well as the IFNγ response resembled that observed in miR-155-deficient mice [78]. Thus, miR-155 plays a dominant role, compared to miR-146a, in T cell-mediated anti-tumor immunity [78].

### 3.5. T Helper 17 Lymphocytes (Th17)

T helper lymphocytes (Th) can be subdivided into three subpopulations: Th1, Th2 and Th17, whose differentiation is driven by IFN-γ, IL-4 and TGF-β/IL-6/IL-21, respectively. Th17 cells are defined by their production of IL-17. These cells play an important role in maintaining mucosal barriers and in favoring pathogen clearance at the mucosal surface; their deregulation is involved in autoimmune and inflammatory disorders. 

The analysis of miR-146a^−/−^ mice showed an expansion of Th17 cells at the level of various segments of the intestine [79], thus implying that miR-146a constrains the number of Th17 cells in the intestine. Furthermore, in some autoimmune conditions, such as immune thrombocytopenia, there is an increase in Th17 cell frequency, associated with a decreased miR-146a expression (in these patients, an inverse relationship between miR-146a levels and Th17 cell frequency was observed) [80].

An initial study by O’Connell and coworkers provided the fundamental observation that miR-155 is required for the development of inflammatory T lymphocytes, including Th17 and Th1 lymphocytes. This effect is related to two different mechanisms, one operating at the level of hematopoietic cell differentiation and another through the stimulation of the production of cytokines favoring Th17 cell differentiation by dendritic cells [81]. miR-155 enhances IL-17 production by the targeting of SOCS1; furthermore, SOCS1 targeting by miR-155 may represent also an additional mechanism through which this microRNA stimulates Th17 cell differentiation [82].

In Th17 cell responses and particularly in *Helicobacter pylori* infection and in mouse inflammatory models, miR-155 was shown to stimulate IL-17 and IFN-γ production. The comparative analysis of WT and miR-155-deficient Th17 cells allowed defining an essential role for JARID2: in fact, miR-155-deficient Th17 cells expressed increased amounts of JARID2, a DNA-binding protein that binds the polycomb repressive complex 2 (PRC2) to chromatin. The increased PRC2 binding to chromatin and H3K27 histone methylation determines the silencing of IL-22, IL-10, IL-9 and ATF3 [71]. JARID2 deletion in part corrects the defects in cytokine production displayed by miR-155^−/−^ Th17 cells [83].

Th17 cells are central to the pathogenesis of autoimmune diseases, and thus, it is not surprising that miR-155^−/−^ mice are resistant to the development of experimental models of autoimmune diseases, such as autoimmune encephalomyelitis. The exploration of this model provided evidence that proper miR-155 expression in Th17 cells is required for Ets1 repression, required for optimal IL-23R expression [84].

## 4. miR-146 and miR-155 in Myeloid Neoplasia

miR-146a and miR-155 expression is deregulated in many neoplasia, where in some cases, there is evidence for a potential pathogenic role. 

Initial studies have shown that miR-155 is an oncogene in B cell lymphoma: its expression is clearly increased in Hodgkin lymphoma and diffuse large B cell lymphoma [13,85]. Transgenic expression of miR-155 in B lymphocytes [86], through targeting and down-modulation of SHIP1 and CEBPβ [87], triggers B cell lymphoma development in mice. In line with these observations, miR-155 downregulation promotes cell cycle arrest and apoptosis in diffuse large B cell lymphoma [88].

miR-155 could act as an oncogene for a set of acute myeloid leukemias (AMLs). Initial studies carried out in a screening on nucleophosmin1-mutated (NPM1 AMLs) have shown a preferential miR-155 overexpression in the subset of these AMLs with FLT3-internal tandem duplication (FLT3-ITD) mutations [89]. Subsequent studies have confirmed the overexpression of miR-155 in AMLs associated with FLT3-ITD mutations [90,91]. Importantly, AMLs overexpressing miR-155 are associated with a poor outcome, as evaluated in terms of complete remission rate, disease-free survival and overall survival [92]. miR-155 high expression in AMLs was associated with a gene expression profile enriched in genes associated with deregulated cellular metabolism or with staminality [93]. Next generation sequencing studies further provided evidence that miR-155 is highly expressed in FLT3-ITD-mutated AMLs, but not in FLT3-TKD-mutated AMLs or in FLT3-WT AMLs. Furthermore, the block of FLT3-ITD-induced miR-155 in vivo significantly reduces the accumulation of leukemic blasts in the bone marrow of mice transplanted with FLT3-ITD^+^ AML cells [94].

Some studies have investigated the possible molecular mechanisms responsible for miR-155 overexpression in AMLs and the possible functional consequences induced in these cells by miR-155 overexpression. Thus, Gerloff and coworkers have shown that the FLT3 signaling induces miR-155 expression, as based on various lines of evidence: FLT3 inhibitors decrease miR-155 expression in FLT3-ITD-mutated cells; FLT3-ITD downstream targets NF-ĸB and signal transducer and activator of transcription 5 (STAT5) regulate miR-155 expression (STAT5 and NF-ĸB bind to the miR-155 gene promoter and stimulate its transcription); inhibition of miR-155 expression blocks the proliferation and induces apoptosis of FLT3-ITD-mutated leukemic blasts [95]. These conclusions were further supported by the observation that the NEDD8-activating enzyme inhibitor MLN4924 inhibits NF-ĸB activation in FLT3-ITD^+^ leukemic cells and, through this mechanism, reduces miR-155 expression, resulting in upregulation on the miR-155 targets (SHIP1 and PU.1), and induces apoptosis [96]. Several studies have shown that the PI3K/AKT SHIP1 phosphatase is a key target of miR-155 in AML cells: the targeting of SHIP1 by miR-155 results in a spontaneous activation of the PI3K/AKT signaling pathway in leukemic cells [97].

Some studies have explored the effect of miR-155 overexpression in leukemic cells on cell differentiation. These studies were carried out using different cellular models and have generated conflicting results. Two studies provided evidence that miR-155 expression stimulates monocytic differentiation in THP-1 [98] or in OC-AML3 cells [99]. However, it must be pointed out that the effects reported in these two studies on cell differentiation are only modest. Opposite conclusions were reached in other studies. Thus, Gerloff et al. have explored the effect of miR-155 expression in monocytic differentiation [95]. Khalife and coworkers showed that the NEDD8 inhibitors lower miR-155 levels and induce differentiation of MV4-11 cells [96]. The reasons for these discrepancies are not easy to understand and may be related to differences in the cellular models used and in the levels of miR-155 expression achieved in these different models. A careful evaluation of this issue, as well as of all of the problems of the role in miR-155 overexpression in leukemia development must be carefully considered. 

Mice transplanted with hematopoietic stem/progenitor cells manipulated to overexpress miR-155 develop a myeloproliferative disorder, not progressing to leukemia [100]. Similarly, FLT3-ITD expression in a murine knockin model resulted in progenitor expansion and a myeloproliferative neoplasm [101]. C/EBPA inactivation is a frequent event occurring in FLT3-ITD AMLs and is responsible for the differentiation block [102]. Therefore, it seemed interesting to evaluate a possible cooperation between miR-155 overexpression and C/EBPA silencing in inducing leukemia development. In line with this hypothesis, ectopic miR-155 expression and loss of C/EBPA expression cooperate in transformation of HSCs/HPCs toward AML, in the absence of FLT3-ITD [103].

The studies performed in various hematological neoplasia, including AMLs and particularly lymphomas, have strongly supported the rationale of using agents able to lower miR-155 levels in vivo as an anticancer strategy. An unconjugated, Locked Nuclei Acid (LNA)-modified oligonucleotide against miR-155 (Mirage-106, MRG-106) was developed by the Mirage Therapeutics, Inc. Company, Boulder (C=), USA and was evaluated in preclinical studies involving AMLs and cutaneous lymphomas: Concerning AMLs, MRG-106 was evaluated in immunodeficient NOD/SCID gamma mice xenografted with a human FLT3-ITD mutated MV4-11 leukemic cell line, showing that its injection elicited a significant enhancement of animal survival [104]. Furthermore, MRG-106 induced apoptosis of primary FLT3-ITD-mutated AML blasts [104].

miR-155 is markedly overexpressed in cutaneous T cell lymphomas (CTCL), and its level of expression together with the other two miRs (miR-203 and miR-205) is of diagnostic value for this disease [105]. Preclinical studies have supported the clinical development of MRG-106 as an experimental drug for the treatment of CTCLs for its pharmacodynamic, pharmacokinetic and safety profile. Thus, a phase I first-in human study was performed to evaluate the safety tolerability, pharmacokinetics and preliminary efficacy of MRG-106 in patients with mycosis fungoides (the most frequent type of CTCLs). The results obtained on five evaluable patients, presented at the last American Society of Hematology (ASH) Meeting, showed that intra-tumor injection of MRG-106 was well tolerated and demonstrated encouraging therapeutic improvements in cutaneous lesions and a significant decrease in systemic disease-related symptoms, such as pruritus [106]. Importantly, on 31 March 2017, the U.S. Food and Drug Administration granted orphan-drug designation to the Mirage product candidate, MRG-106.

miR-146a expression was initially investigated in primary AML samples, subdivided according to the French American British (FAB) classification. Spinello and coworkers provided evidence that miR-146a was expressed at lower levels in AMLs with a more mature morphological phenotype (M3, M4 and M5 AMLs), compared to AMLs with a more immature phenotype (M1 and M2 AMLs) [107]. Similarly, Lutherborrow et al. observed that both miR-146a and miR-146b are markedly less expressed in M5 than in M1 AMLs [108]. Importantly, more recent studies showed that a low miR-146a expression level in AMLs identifies an AML subset with a reduced survival, compared to those with high miR-146a levels [109]. Interestingly, these authors observed also that AML patients displaying a higher increase in miR-146a levels following pre-treatment with G-CSF before low-dose standard chemotherapy showed a better overall survival and higher complete remission rates [110].

These studies showed that CXCR4 and Smad4 may represent important targets of miR-146a in AML cells. Particularly, Spinello and coworkers showed that in primary AMLs, there is an inverse correlation between miR-146a levels and CXCR4 levels [107]; furthermore, miR-146a overexpression in leukemic cell lines down-modulates CXCR4 expression [107,109]. CXCR4 down-modulation induced by miR-146a overexpression may be used as a strategy to reduce the stromal-induced chemoresistance [108]. Interestingly, G-CSF administration induces a miR-146a up-modulation responsible for a reduction of CXCR4 and lower chemoresistance [110]. 

These studies did not show a link between a deregulated (low) miR-146a expression and peculiar molecular abnormalities of leukemic blasts. Li and coworkers have investigated the miRNA expression profiles in AMLs with common translocations and observed that miR-146a is specifically down-modulated in AMLs with Mixed Lineage Leukemia (MLL) rearrangements, but not in other frequent AML-associated translocations [111]. In a recent study, the molecular mechanisms responsible for miR-146a down-modulation observed in MLL-rearranged AMLs have been explored. MEIS1 and HOXA9 are two key target genes of MLL-fusion proteins: their overexpression is required for the induction and maintenance of MLL-rearranged AMLs. MEIS1/HoxA9 down-modulate PU.1 expression, and this in turn reduces miR-146a expression; the low miR-146a expression leads to an increase of SYK expression and phosphorylation (SYK was shown to be a direct target of miR-146a) [112]. The SYK up-modulation triggered through this miR-146a-dependent mechanism was found to be an event fundamental for leukemic transformation mediated by MLL-rearranged genes [112].

As mentioned above, a reduced miR-146a expression is in part responsible for increased CXCR4 expression in AMLs; this condition supports the leukemogenic effect of MEIS1 in that MEIS1 promotes transactivation of synaptotagmin-like 1 protein, involved in the activation of the CXCL12/CXCR4 axis [113].

Abnormal miR-146a expression plays an important pathogenic role in some myelodysplastic syndromes (MDSs). The most frequent chromosome abnormality observed in MDSs consists of a deletion of chromosome 5q (delOK(5q)). MDS patients with a del(5q) are characterized by some phenotypical features, including refractory anemia, neutropenia and thrombocytosis, associated with megakaryocytic dysplasia (hypolobulated megakaryocytes). The commonly-deleted regions of chromosome 5 involve also the loci of two miRs, miR-145 and miR-146a. The deletion of these two miRs contributes in a relevant way to the pathogenesis of these MDSs, being responsible for thrombocytosis associated with hypolobated megakaryocytes, neutropenia and clonal dominance [114]. As is expected, studies performed in bone marrow mononuclear cells, as well as CD34^+^ cells, isolated from del(5q) MDS patients, have shown a reduced expression by approximately half, in line with the deletion of a single allele (reviewed in [115]).

Several studies have in part clarified the mechanism(s) through which miR-146a deficiency contributes to MDS development in del(5q) patients. An important molecular consequence of del(5q) is an increased expression of TRAF6, resulting from miR-146a haploinsufficiency [114]. Importantly, TRAF6 overexpression in mouse Hematopoietic Stem Cells/Hematopoietgic Progenitor Cells (HSCs/HPCs) resulted in several defects of the hematopoietic system, including neutropenia, dysplasia and elevated platelets: neutropenia and thrombocytosis seem to be related to increased IL-6 production [114]. It is important to note the similarity of the phenotype of TRAF6 overexpressing mice with the phenotype of mIR-146a-deficient mice, except for platelet abnormalities absent in the miR-146a-deficient mice. However, TRAF6-overexpressing mice exhibit TRAF6 levels about 10-times higher than those observed in del(5q) cells [115]. Thus, other studies were focused on characterizing the role of additional genes deleted in del(5q) MDSs. Another gene constantly deleted in del(5q) MDSs is the TRAF-interacting protein with forkhead-associated domain B (TIFAB); TIFAB gene haplo-insufficiency may contribute to the MDS phenotype through two different mechanisms: TIFAB acts as a repressor of TRAF6 since it forms an unstable complex with this protein, reducing TRAF6 protein stability; TIFAB-deficient mice display a number of defects of hematopoiesis, including an altered number of HSCs/HPCs, impaired myeloid differentiation and progressive cytopenia [116]. In experimental mouse models, miR-146a deficiency and TIFAB deficiency synergistically cooperate in inducing hematopoietic abnormalities, through a process of epistatic cooperation [117].

## 5. Conclusions

miR-146a and miR-155 have many similarities in the large spectrum of their biologic activities under normal and pathologic conditions. Both of these two miRNAs are expressed and function in a variety of immune cell types, including monocytes/macrophages, dendritic cells, Natural Killer (NK) lymphocytes, various T cell subsets and B lymphocytes. The fine tuning of miR-146a and miR-155 expression in these cells is essential for an appropriate control of various essential immune functions, and usually, these two miRNAs act in an opposite way.

Various functional studies have directly supported the key physiological role of these two miRs in the control of immune response. Furthermore, the functional role of their target genes was also carefully documented. In this context, an elegant approach consisted of the genetic disruption of the RNA binding site in the 3′UTR of an miR-155 target gene, SOCS1, which phenocopied many of the T- and NK cell defects observed in mice lacking miR-155 [118].

A disease-related expression of miR-146a and miR-155 contributes to the pathogenesis of various pathologic conditions related to pathogen infections or associated with excessive immunological reactivity (allergic diseases, autoimmunity diseases). There is evidence, in some of these conditions, that the targeting of miR-146a or of miR-155 may represent a potential therapeutic tool.

Furthermore, the expression of miR-146a or miR-155 is deregulated in many tumors. Particularly, the expression of these two miRNAs is deregulated in some mammalian hematopoietic cancers: miR-155 is markedly overexpressed in some lymphomas and in FLT3-ITD-mutated AMLs, where there is evidence that this miR acts as an oncomiR; miR-146a is clearly down-modulated in some AMLs and in del(5q) MDSs. The findings observed for miR-155 have promoted the development of inhibitory-miRNA therapies based on antisense antimiRs. Thus, MRG-106, a synthetic microRNA antagonist of miR-155, was developed and tested with preliminary promising results in patients with cutaneous T cell lymphoma of the mycosis fungoides subtype. Future applications are expected using miR-155 antagonists in other tumors and, possibly, also in some non-neoplastic conditions. The same could apply for the possible introduction in clinical experimentation of miR-146a mimetics.

Thus, the studies carried out in these last few years on the expression and function of miR-146a and miR-155 in normal and pathological conditions underline the importance of basic research studies on the biological activities of non-coding mi-RNAs and on their targeting for therapeutic purposes.
